# Mimicking the LOX-Related Autosomal Recessive Congenital Ichthyosis Skin Disease Using a CRISPR-Cas9 System and Unravelling 12S-LOX Function in the Skin

**DOI:** 10.3390/dermatopathology12030030

**Published:** 2025-09-11

**Authors:** Carolyne Simard-Bisson, Sébastien Larochelle, Véronique J. Moulin, Bernard Fruteau de Laclos

**Affiliations:** 1 Centre de Recherche du CHU de Québec-Université Laval and Centre de Recherche en Organogénèse Expérimentale de l’Université Laval/LOEX, Quebec, QC G1J 1Z4, Canada; carolyne.simard-bisson@crchudequebec.ulaval.ca (C.S.-B.); bernard.fruteau-de-laclos@fmed.ulaval.ca (B.F.d.L.); 2 Department of Surgery, Faculty of Medicine, Université Laval, Quebec, QC G1V 0A6, Canada; 3 Department of Molecular Biology, Medical Biochemistry and Pathology, Faculty of Medicine, Université Laval, Quebec, QC G1V 0A6, Canada

**Keywords:** epidermis, keratinocytes, lipoxygenase, tissue engineering, CRISPR-Cas9 systems

## Abstract

*Stratum Corneum* (SC) formation in the human epidermis requires lipid processing. Lipoxygenases (LOXs) such as 12R-Lipoxygenase (12R-LOX) and Epidermis-type lipoxygenase 3 (eLOX-3) contribute to this process. Mutations in their genes cause Autosomal Recessive Congenital Ichthyosis (ARCI) in patients. On the other hand, 12S-lipoxygenase (12S-LOX) is expressed in the human epidermis, but its role still remains to be clarified. The involvement of eLOX-3, 12R, and 12S-LOX in conditions or processes such as skin photodamage, wound healing, psoriasis, and atopic dermatitis is suggested but still remains unclear. In order to eventually gain a better understanding of the role of these LOXs in such processes, models of Tissue-Engineered Skins (TESs) with an impaired expression for the native form of either eLOX-3, 12R-LOX, or 12S-LOX were produced using CRISPR-Cas9(D10A) technology. All three models showed impaired keratinocyte differentiation and changes in the prevalence or the size of lipid droplets within the most superficial layers, thus reproducing features observed in ARCI and supporting a role for 12S-LOX in SC formation. Since eLOX-3 and 12R-LOX depleted TES’s reproduced features observed in ARCI, such models can be considered as reliable tools for the functional studies of these LOXs in the human epidermis.

## 1. Introduction

Skin barrier function requires *Stratum Corneum* (SC) formation [1]—a process achieved through keratinocyte differentiation into corneocytes [1,2]. During this process, lipids are secreted by keratinocytes and assembled into the Cornified Lipid Envelope (CLE) [3,4,5,6]. The involvement in the CLE assembly of eLOX-3 and 12R-LOX [4] (enzymes, respectively, encoded by the ALOXE3 and ALOX12B genes) was considered because mutations within these genes are associated with the Non-bullous Congenital Ichthyosiform Erythroderma (NCIE) [7]. This inherited keratinisation disorder (belonging to the Autosomal Recessive Congenital Ichthyoses—ARCI) causes abnormal skin scaling, diffuse redness, and reduced skin barrier function [8,9]. In knockout mouse models for 12R-LOX and eLOX-3, altered lipid profiles and increased transepidermal water loss (TEWL) have been observed [10,11,12,13].

For 12S-LOX, no inherited disease has been associated, to date, with mutations in the gene responsible for its encoding (ALOX12). However, a slight increase in TEWL has been described in an Alox12 knockout mouse model [14]. Therefore, the involvement of 12S-LOX in SC formation is suspected but still uncertain.

A role for eLOX-3, 12R, or 12S-LOX in conditions or processes such as psoriasis [15,16,17,18,19], skin photodamage [20], atopic dermatitis [21,22], inflammation, or wound healing [18,23,24,25] is suggested but still remains to be clarified. Therefore, the goal of this study was to create a human skin model showing reduced levels of eLOX-3, 12R, or 12S-LOX in the epidermis in order to study the role of these LOXs in a variety of processes in the skin.

Here, we took advantage of the fusion of two technologies—gene editing via double nicking using a lentiviral-delivered RNA-guided CRISPR-Cas9(D10A) system and human Tissue-Engineered Skin (TES). TES with a reduced expression of the native form of eLOX-3, 12R-LOX, or 12S-LOX (CRISPR-LOX TES) generated in this study showed a granular surface, an altered SC, and lipid droplets increasing in prevalence or in size. Our model successfully reproduced some features seen in eLOX-3- or 12R-LOX-related ARCI or in knockout mouse models, thus confirming its reliability. To our knowledge, this study reports a first human model of 12S-LOX native form depletion, revealing impaired keratinocyte differentiation and thus supporting a role for this enzyme in this process. The CRISPR-LOX TESs shown here represent interesting models for future studies on the involvement of LOXs in various processes within the human epidermis.

## 2. Materials and Methods

### 2.1. Production of CRISPR-Cas9(D10A), Lentiviral Vector Production, and Cell Culture

See the Supplementary Materials section for details [26,27,28,29,30].

### 2.2. Skin Biopsies

After informed consent was obtained, healthy human skin specimens were obtained following facelift or breast resection surgery.

### 2.3. Production of CRISPR-LOX Plasmids

Non-targeting control sgRNAs or two separate sgRNAs targeting either eLOX-3, 12R, or 12S-LOX were designed for the nickase Cas9(D10) system using the CRISPR sgRNA design platform from ATUM (www.atum.bio). To induce mutations that interfere with the enzymes’ activity, sgRNAs were selected to disrupt the gene sequence before the iron-binding domain of the different LOX genes. The specificity of each sgRNA was evaluated using the BLAST tool version 2.9.0 from the National Library of Medicine [31] and CRISPOR [32]. Selected sgRNA sequences, specificity scores, and the number of off-targets near a PAM site are indicated in Table S1, whereas possible off-target genes are indicated in Table S2. Single-stranded oligonucleotides encoding the variable sequence of the sgRNAs were synthesised by Integrated DNA Technologies, annealed to form double-stranded DNA with cohesive ends, and inserted into an sgRNA scaffold downstream of either the H1 promoter (phH1-gRNA was a gift from Charles Gersbach; Addgene plasmid # 53186; http://n2t.net/addgene:53186; RRID:Addgene_53186, accessed on 9 August 2025) or the 7SK promoter (ph7SK-gRNA was a gift from Charles Gersbach; Addgene plasmid # 53189; http://n2t.net/addgene:53189; RRID:Addgene_53189, accessed on 9 August 2025). For sgRNA ligation, plasmids were digested using the Bbs1-HF restriction enzyme (New England Biolabs, Ipswich, MA, USA, #R35395) and ligation was performed using T4 DNA ligase (New England Biolabs #M0202L) according to the manufacturer’s instructions. The resulting constructions were amplified using One Shot Stbl3 chemically competent E. coli (Invitrogen, Waltham, MA, USA, #C737303), as suggested by the manufacturer. Golden Gate assembly of the resulting constructions with the modified pLV hUbC-Cas9(D10A)-T2A-GFP was performed as described [33] except that the missing cassettes (hu6 and mu6) were replaced by a DNA adapter (sequence in Table S2) at a molar concentration 4 times higher than the one used for the plasmids. Following Golden Gate assembly, SURE2-competent cells (Agilent, Santa Clara, CA, USA, #200152) were transformed with the resulting plasmids. After the selection and amplification of positive colonies, plasmid DNA was extracted using the EZ-10 spin column plasmid DNA miniprep kit (Bio Basic, Markham, Canada #BS413). The insertion of the sgRNA in the resulting constructions was confirmed by sequencing with the primers listed in Table S3. The production of lentiviral vectors was then performed as described [34] (see Supplementary Material for details).

### 2.4. Keratinocyte Transduction, Cell Sorting, and Gene Editing Analysis

A total of 60,000 keratinocytes were seeded on a feeder layer of irradiated dermal fibroblasts in 6-well plates and were allowed to adhere overnight in keratinocyte culture medium. The next day, the cell culture medium was removed and replaced by 450 to 600 µL of lentiviral vector solution supplemented with 8 µg/mL polybrene (Sigma-Aldrich, St-Louis, MO, USA, #H9268) in order to achieve an approximate Multiplicity of Infection of 20 MOI/cell. Cells were incubated with lentiviral vectors for one hour at 37 °C. Fresh keratinocyte culture medium was then added to the cells. Cell culture medium was changed three times per week, and the cells were cultured for approximately 10 days in order to allow GFP expression. Then, the most (10%) fluorescent GFP-positive cells were sorted using the FACSMelody cell sorter (Becton and Dickinson, Franklin Lakes, NJ, USA). These transduced cells were either frozen/thawed and/or amplified for another passage before seeding on TES.

### 2.5. PCR and Mutation Analysis

Following cell sorting, DNA was extracted using the DNeasy Blood and Tissue Kit (Qiagen, Germantown, MD, USA, cat. #69504) according to manufacturer’s instructions. Polymerase Chain Reaction (PCR) was performed with the primers specified in Table S3 using Q5 high-fidelity DNA polymerase (New England Biolabs, cat. #M0491S) according to the manufacturer’s indications. The resulting DNA was sequenced and the results were analysed for mutations using the Synthego ICE analysis platform [35].

### 2.6. Production of TES

The production of CRISPR-LOX TESs was performed in two to four independent experiments depending on the type of LOX. TESs were produced as previously described with modifications [36] (see Supplementary Materials for details). Samples were harvested after 14 or 15 days of culture at the air–liquid interface.

### 2.7. Staining and Immunofluorescence Analysis

For Masson’s trichrome staining, TES biopsies were fixed with fresh 4% paraformaldehyde, embedded in paraffin, and cut in 5 µm sections using an RM2245 microtome (Leica, Concord Ontario, ON, Canada). The fixation of the samples for GFP expression studies was performed by dehydrating the samples in a 10% sucrose solution for 2 h and then for 16h in a 30% sucrose solution. For GFP observation, as well as for immunofluorescence and Oil Red O (ORO) staining, samples were embedded and quickly frozen in Tissue-Tek OCT compound (Sakura Finetek, Torrance, CA, USA, #4583). Samples were cut in 6 to 8 µm thick sections. For GFP observation, samples were dried for 20 min and then directly observed. For LOX staining, cryosections were fixed as previously described [37,38]. Staining for transglutaminase 1 and filaggrin was performed on tissue sections treated for 10 min in 100% acetone at –20 °C. This treatment induced the loss of the GFP present in the cells, thus allowing the subsequent use of the green channel. The antibodies used for immunofluorescence staining are listed in Table S4 [39]. In all immunofluorescence staining, nuclei were stained by adding Hoescht 33258 (Sigma-Aldrich, #B2883) at a concentration of 0.5 µg/mL to the secondary antibody. For ORO staining, sections were fixed for 10 min in cold 3.7% formalin. After 3 washes in water, slides were incubated for 2 to 5 min in an 85% solution of 1,2-propanediol (Sigma-Aldrich, #P6209). Samples were then stained by immersing the slides in a 0.25% solution of ORO (Sigma-Aldrich, #O06225) diluted in 100% 1,2-propanediol for 10 min at 60 °C. Slides were then incubated for 2 min in 85% 1,2-propanediol before being washed twice in water and mounted. Pictures were acquired as previously described [28].

### 2.8. Ultrastructural Analysis

Samples were prepared as previously described [40]. Transmission electron microscopy (TEM) pictures were acquired using an FEI Tecnai Spirit G2 device (ThermoFisher Scientific, Waltham, MA, USA) operating at 80 kV and coupled with a Hamamatsu ORCA-HR digital camera (10 MP).

### 2.9. Statistical Methods

Statistical analyses were performed using data collected in two independent experiments, in which each condition had 2 to 4 replicates. For each sample, the thickness of the living epidermis (from the basal to the granular layer) was measured at 4 different points on TES cryosections using ImageJ (version 1.53e). For cell proliferation analysis, cryosections stained with Hoescht and anti-Ki67 antibody were used to quantify the number of nuclei and of Ki67-positive cells in the basal layer. For each section, the ratio of the number of Ki67-positive cells to the total number of nuclei in the basal layer was determined. The mean of these ratios and the standard variations were calculated for each condition and used to produce the presented graph. To analyse the parakeratotic cells in the most superficial layers of the epidermis, cryosections stained for nuclei with Hoescht were used; quantification was performed for each field per sample. All graphs show the resulting ratio of the median for a group divided by the median of the non-transduced control, as well as the first and the last quartile and the range of the data. The ratio for the non-transduced control is indicated by a dotted line. For statistical analysis, a rank comparison test (Kruskall–Wallis) between each condition and the controls (non-transduced and non-targeting sgRNA control TESs) was performed with a *p* < 0.05. Analyses were performed using GraphPad 10.

## 3. Results

### 3.1. Transduction and Enrichment of Keratinocytes Depleted in the Native Form of Either eLOX3, 12R-, or 12S-LOX Using a CRISPR-Cas9(D10A) Lentiviral System

**Figure 1:**
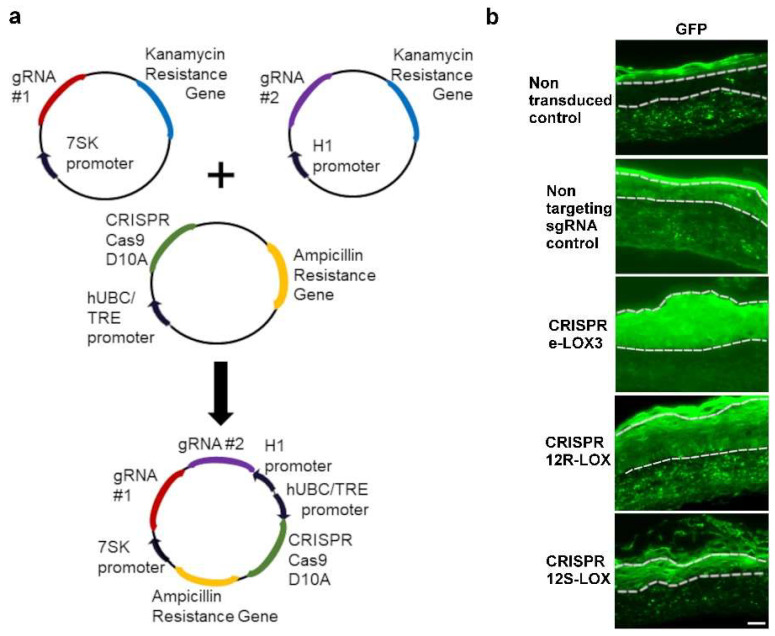
Production of CRISPR-LOX TESs. (**a**) Plasmid assembly diagram. sgRNAs were inserted into a 7SK- or H1-promoter cassette. H1, 7SK, and CRISPR-Cas9(D10A)-GFP cassettes were combined using Golden Gate assembly. (**b**) Green fluorescence associated with the presence of the CRISPR-Cas9(D10A) system in tissue sections of the TESs. Dotted lines delineate the living epidermis. Scale bar: 50 µm.

To permanently impair the expression of the native forms of eLOX-3, 12R-LOX, and 12S-LOX in keratinocytes, we selected and modified a CRISPR-Cas9 genome editing system originally developed by Kabadi et al. [33] to enable double nicking using the Cas9(D10A) mutant. Such a variant of the Cas9 enzyme has the advantage of increasing gene editing efficiency, as well as reducing off-target effects to indetectable levels [41]. The sgRNA was also selected for its high specificity and low off-target effects (see Table S2). To ensure LOX loss of function, three loci per gene were tested, each requiring two sgRNAs that bind opposite DNA strands in close proximity. The selected sgRNA pairs, either targeting the LOX genes of interest or serving as non-targeting controls (not matching any sequence within the human genome—see [42]), were cloned into either the H1 or the 7SK expression cassettes [33]. A Golden Gate assembly was then performed to combine these sgRNA-expressing cassettes with the pLV hUbC-Cas9(D10A)-T2A-GFP plasmid (Figure 1a). The proper assembly was confirmed by sequencing. The resulting construct was then used to generate lentiviral vectors. After transduction with the lentiviral vectors, keratinocytes were sorted by FACS based on their GFP expression to specifically select the transduced cells. At the final stage, sgRNA pairs for which cell proliferation was successful and showing the highest indel frequency were selected. Indel frequency was determined by extracting, sequencing, and analysing keratinocyte DNA using the ICE CRISPR analysis tool (ICE v3) [35] (see Figures S1–S3). This software compared non-modified DNA (here, DNA from the cells transduced with the non-targeting sgRNA) with gene-edited DNA, detecting the differences between these DNA sequences. It revealed an indel ratio of 85% for eLOX-3, 93% for 12R-LOX, and 49% for 12S-LOX genes. Such an indel ratio was considered high enough to induce a reduction in the expression of the native form of the different LOXs in the TES model by generating a frameshift that would most likely result in the creation of nonsense or missense mutations in the selected LOX genes.

Previously selected GFP-expressing keratinocytes were then used to produce TESs—a model in which these LOXs are naturally expressed [38]. Keratinocytes were seeded on tissue-engineered dermal sheets, allowed to grow for 4 days, and placed at the air–liquid interface to induce their differentiation. To confirm the persistence of the introduced sgRNA-guided CRISPR-Cas9(D10A) system in keratinocytes, the presence of GFP was investigated in the epidermis of the TES after 14 days of culture at the air–liquid interface (Figure 1b). As expected, green fluorescence was observed within the SC and in the dermis of the non-transduced TESs. This signal is due to the autofluorescence naturally present in these parts of the tissue [43,44]. Since the dermis and the SC produce autofluorescence, the observation of green fluorescence to confirm GFP expression must be limited to the living layers of the epidermis (basal to granular layers). As anticipated, green fluorescence was absent from the living epidermis (section between dotted lines Figure 1b) in the TESs produced with non-transduced keratinocytes. TESs produced with transduced keratinocytes, however, showed green fluorescence within the entire living epidermis. This confirmed the appropriate selection, growth, and preservation of the transduced keratinocytes (Figure 1b).

To confirm the reduced expression of the native LOXs within the various TESs, immunofluorescence staining of eLOX-3, 12R-LOX, and 12S-LOX was performed. In non-transduced and non-targeting sgRNA controls, the different LOXs were observed at the cell periphery of keratinocytes from the basal to the granular layer, as previously described [38].

**Figure 2:**
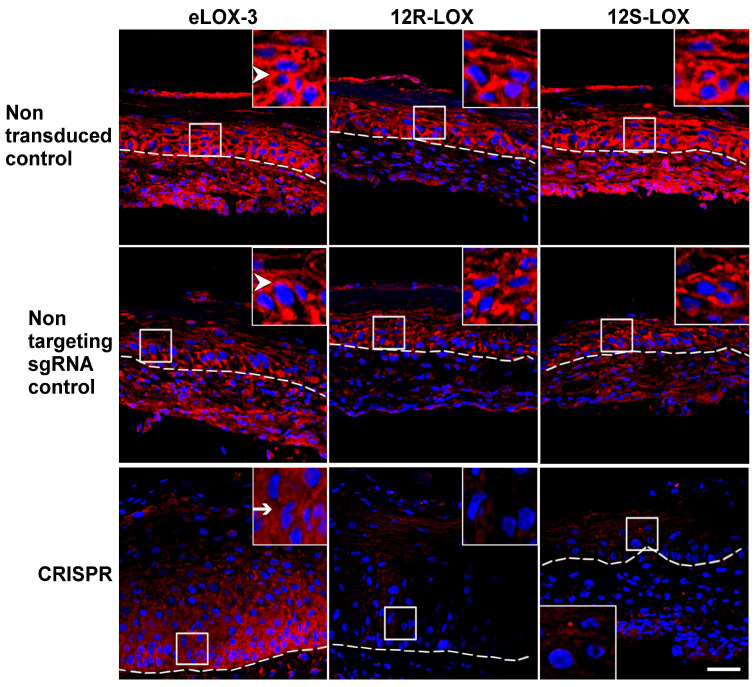
eLOX-3, 12R-LOX, and 12S-LOX immunofluorescence staining in non-transduced, non-targeting sgRNA control, CRISPR eLOX-3, CRISPR 12R-LOX, or CRISPR 12S-LOX TESs. Dotted lines delineate the dermoepidermal junction. The small squares delineate the magnified area shown in the top right or bottom left corner of each picture. Note that LOX staining (strong red staining at the cell periphery; see arrowheads) is absent in CRISPR-LOX TESs. Non-peripheral red staining (arrow) in CRISPR eLOX-3 corresponds to non-specific signals. Nuclei are in blue. Scale bar: 50 µm.

As expected, the expression of the native LOXs was markedly reduced in their corresponding CRISPR-LOX TESs (Figure 2). These results confirmed a decrease in native eLOX-3, 12R-LOX, or 12S-LOX expression within the various models.

### 3.2. Impact of the Reduced Expression of the Native Forms of the Selected LOXs on TES Phenotype and Cell Proliferation

**Figure 3:**
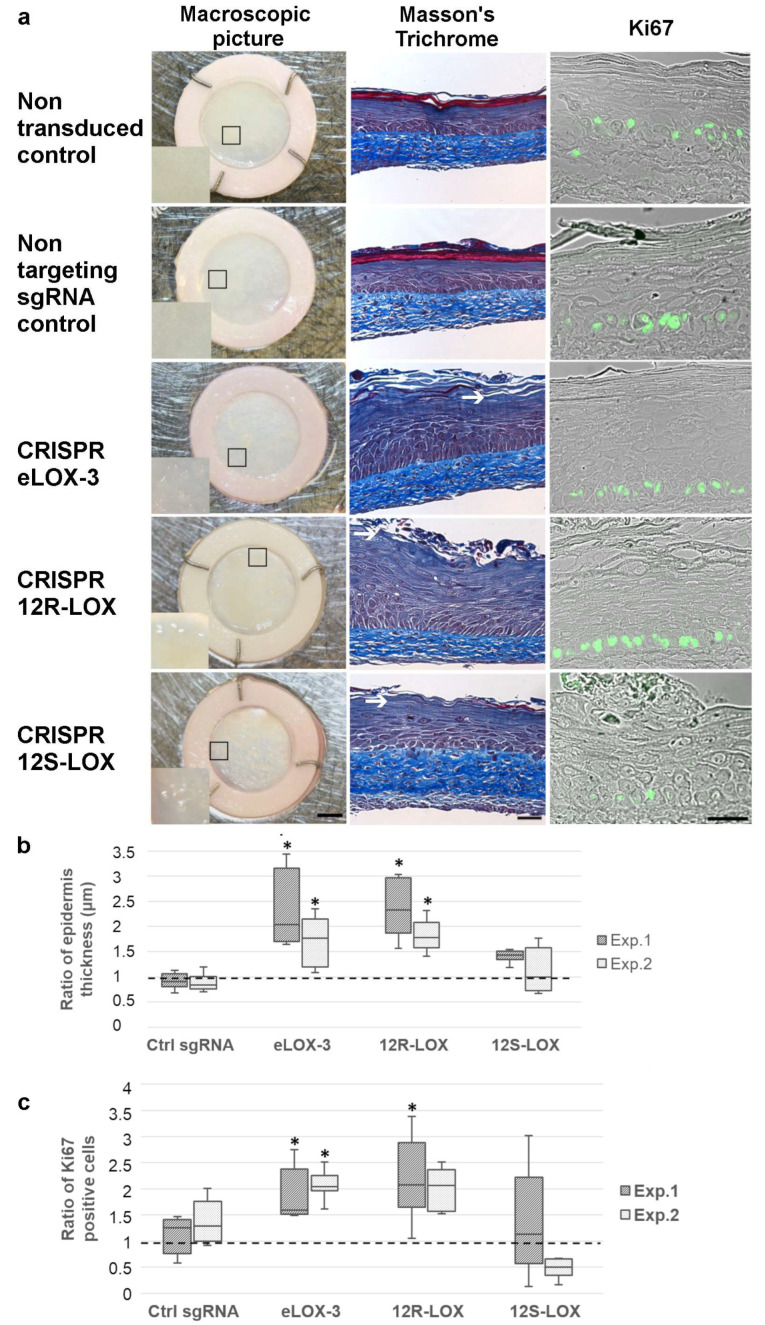
Aspect and epidermal cell proliferation in the TESs. (**a**) Macroscopic pictures: Masson’s trichrome and Ki67 staining (green) combined with phase contrast pictures of non-transduced, non-targeting sgRNA, CRISPR eLOX-3, CRISPR 12R-LOX. and CRISPR 12S-LOX TESs. The small squares delineate the magnified area shown in the bottom left corner of the TES macroscopic pictures. Arrows indicate deficient SC. Scale bars: 2 mm (macroscopic pictures); 50 µm (Masson’s trichrome and Ki67 staining). (**b**) Ratios of epidermal thickness (in µm) and (**c**) Ki67-positive cells in the basal layer of the different TESs compared to the median of the non-transduced control (dotted line) for experiment 1 (dark colour) and experiment 2 (light colour). Graphs indicate the median (bar in the centre of the box), the first and last quartile (lower and upper part of the box), and the data range (line). Asterisks indicate significant results using a rank comparison test (Kruskall–Wallis) when comparing each condition to both the non-transduced and the non-targeting sgRNA control TESs (*p* < 0.05).

In order to investigate the impact of the depletion of the native form of the various LOXs selected for this study, macroscopic pictures of each TES were taken on day 14 of air–liquid interface culture. Non-transduced and non-targeting sgRNA control TESs showed a rather regular and smooth epidermis, whereas CRISPR-LOX TESs showed a granular surface, suggesting a disorder within the keratinocyte differentiation process (Figure 3a). To investigate this phenotype more deeply, tissue sections were stained with Masson’s trichrome. Non-transduced and non-targeting sgRNA control TESs showed normal keratinocyte differentiation, resulting in proper SC formation. On the other hand, the epidermis of the various CRISPR-LOX TESs displayed an absent or altered SC, thus suggesting a defect in keratinocyte differentiation or SC fragility (Figure 3a).

Since the living epidermis of eLOX-3 and 12R-LOX CRISPR-LOX TESs appeared thicker on tissue sections, a statistical analysis of the living epidermis thickness (measured from the basal to the granular layer) was performed. The living epidermis of CRISPR eLOX-3 and CRISPR 12R-LOX TESs was significantly thicker when compared with the controls (Figure 3b).

To determine whether the increase in the living epidermis thickness was due to higher levels of cell division, cell proliferation was assessed. This was performed by staining the various TESs for Ki67 (a marker of dividing cells). As shown in Figure 3a,c, when compared with the non-targeting sgRNA control, the proportion of Ki67-positive cells within the basal layer appeared to be higher in CRISPR 12R-LOX and CRISPR eLOX-3 TESs.

In summary, CRISPR eLOX-3 and CRISPR 12R-LOX TESs showed similar phenotypes (an altered SC with a thicker living epidermis), whereas CRISPR 12S-LOX TES only presented defects in the SC.

### 3.3. Impact of the Depletion of the Native Form of the Targeted LOXs on Keratinocyte Differentiation in TES

**Figure 4:**
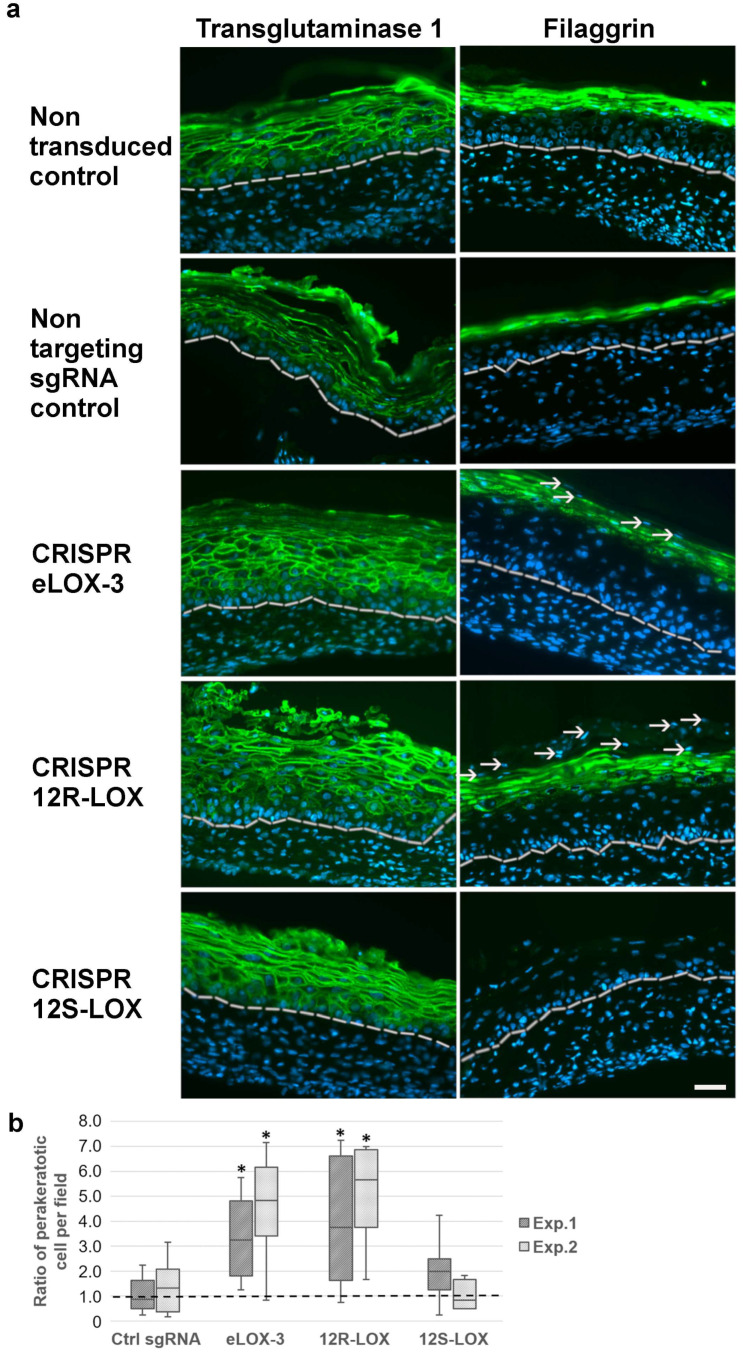
Study of keratinocyte differentiation in control and CRISPR-LOX TESs. (**a**) Immunofluorescence staining pictures for transglutaminase 1 (green) and filaggrin (green) in non-transduced, non-targeting sgRNA, CRISPR eLOX-3, CRISPR 12R-LOX, or CRISPR 12S-LOX TESs. Nuclei are stained in blue. White arrows indicate parakeratotic cells (persistence of nuclei) in the upper layers, while white dotted lines show the dermoepidermal junction. Scale bar: 50 µm. (**b**) Ratios of the number of parakeratotic cells per field in the upper layers of the different TESs compared to the median for the non-transduced control (dotted line) for experiment 1 (dark colour) and experiment 2 (light colour). Graph indicates the median (bar in the centre of the box), the first and last quartile (lower and upper part of the box), and the data range (line). Asterisks indicate significant results using a rank comparison test (Kruskall–Wallis) when comparing each condition to both the non-transduced and the non-targeting sgRNA control TESs (*p* < 0.05).

Because the epidermis seemed to be altered in CRISPR-LOX TESs, keratinocyte differentiation was more deeply assessed in these models. This was achieved using immunofluorescence staining for markers of the spinous (transglutaminase 1) and the granular (filaggrin) layers. Transglutaminase 1 expression was observed at the periphery of keratinocytes in the suprabasal layers, as previously described in TESs [38]. This pattern was consistent across all the models (Figure 4a), suggesting that keratinocyte differentiation at the level of the spinous layer was not altered by the reduction in the native form of the different LOXs.

In the CRISPR 12S-LOX TESs, the expression of filaggrin was not detected, while in the CRISPR eLOX-3 and CRISPR 12R-LOX TESs, filaggrin expression was not modified when compared with the controls [45] (Figure 4a). However, the persistence of cells with a nucleus (parakeratotic cells) was observed in the most superficial layers of the CRISPR eLOX-3 and CRISPR 12R-LOX TESs (Figure 4a). To further address this question, an evaluation of the parakeratotic cells within the most superficial layers was performed. As shown in Figure 4b, the number of parakeratotic cells was significantly higher in CRISPR eLOX-3 and CRISPR 12R-LOX TESs. Such a feature further supported the presence of an impaired keratinocyte differentiation and is in accordance with previous observations in eLOX-3 or 12R-LOX-depleted models [10,46]. In summary, these LOX-depleted models showed an impaired keratinocyte differentiation whether by reduced filaggrin expression in CRISPR 12S-LOX TESs or by increased levels of parakeratotic cells in the CRISPR eLOX-3 and CRISPR 12R-LOX TESs.

### 3.4. Lower Levels of the Native Form of the Targeted LOXs in TES Affects Lipid Droplets and Cell Ultrastructure

**Figure 5:**
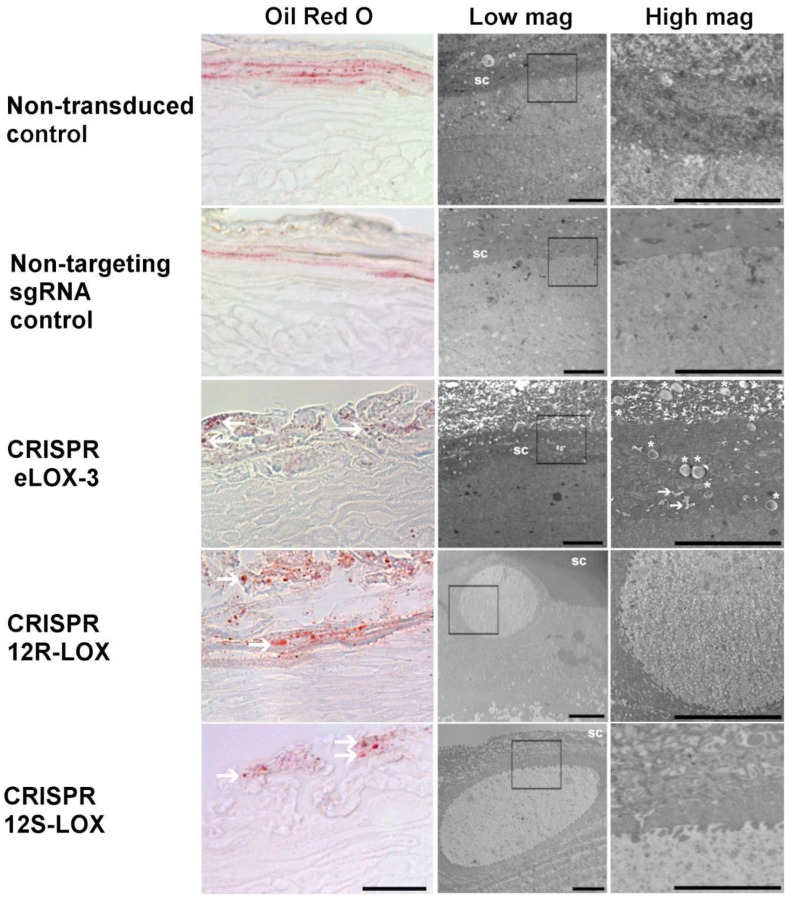
Study of lipid droplets in TESs. ORO staining and low- and high-magnification TEM pictures of non-transduced, non-targeting sgRNA, CRISPR eLOX-3, CRISPR 12R-LOX, and CRISPR 12S-LOX TESs. White arrows and asterisks indicate larger lipid droplets in CRISPR-LOX TESs. Squares delineate the area shown in the picture on the right. SC indicates the stratum corneum. Scale bar: 50 µm (ORO) or 4 µm (low mag. and high mag.).

Since LOXs are well known for their involvement in lipid processing, Oil Red O (ORO) lipid staining was performed on control and CRISPR-LOX TES samples. ORO has the property of staining neutral fats and cholesteryl esters but not the cell membranes [47,48]. ORO staining in the non-transduced and non-targeting sgRNA control TESs showed an abundance of very-small red dots in the most superficial layers of the epidermis (Figure 5). However, this pattern looked particularly different in the CRISPR-LOX TESs. Indeed, ORO staining in eLOX-3 showed medium-sized droplets, whereas larger ORO-stained droplets were seen in CRISPR 12R-LOX and CRISPR 12S-LOX TESs (Figure 5). Such structures could not be seen with Masson’s trichrome staining, probably because of the use of organic solvents during histological processing that may dissolve lipids [49].

As ORO staining suggested the presence of large lipid deposits in CRISPR 12R-LOX and CRISPR 12S-LOX TESs, these deposits were further studied using transmission electron microscopy (TEM). Figure 5 (centre and right panels) shows the ultrastructure of the epidermis near the granular layer and the SC junction for the different TESs. Non-transduced and non-targeting sgRNA control TESs did show a regular granular layer and SC. However, all of the CRISPR-LOX TESs showed corneocytes characterised by a translucent and heterogeneous content, as well as multiple droplets for CRISPR eLOX-3 TES (Figure 5, asterisks and arrows) or large droplets for CRISPR 12R-LOX and CRISPR 12S-LOX TESs. It therefore appears that the reduced expression of the native form of the targeted LOXs induces a modification in the prevalence or the size of lipid droplets in keratinocytes.

## 4. Discussion

In this study, human skin models with a reduced expression of the native forms of either eLOX-3, 12R-LOX, or 12S-LOX within the epidermis were produced. In the literature, a 50% to 80% reduction in the targeted gene is generally obtained in human primary keratinocytes when CRISPR-Cas9 transfection is used [50,51]. These levels of knockdown, leading to mosaic editing, may seem low but are generally sufficient to induce an altered phenotype [50,51]. It is known that knockdown is generally harder to achieve in primary keratinocytes compared to immortalised cell lines [52,53]. Such a difference can be explained in part by the fact that primary keratinocytes have an antiviral mechanism conferring resistance to CRISPR-Cas9 transfection [54,55,56]. However, the indel ratios of 49 to 93% indel obtained in this study were sufficient to induce a phenotype in the TES model, similar to what is seen in other models, and to induce a reduction in LOX staining. The advantage of using a TES model is that it is of human origin and that complete keratinocyte differentiation and LOX expression are present, thus allowing for a clear observation of the impact of LOX depletion on the final stages of skin maturation.

As mentioned, CRISPR eLOX-3 and CRISPR 12R-LOX TES models shared many features with previously described models showing a reduced expression of these LOXs, thus supporting the quality of our model. In both CRISPR eLOX-3 and CRISPR 12R-LOX TESs, a thicker epidermis [10,57], as well as parakeratosis and a trend for increased cell proliferation, were observed [10,12,46,58], thus supporting eLOX-3 or 12R-LOX depletion in those models. It should be noted that it was not possible to observe a thicker SC as it is usually observed in ARCI [59] for CRISPR eLOX-3 and CRISPR 12R-LOX. However, remnants of very-thin SC could sometimes be observed in CRISPR-LOX TESs, thus suggesting that SC formation did take place but that it could not be maintained, maybe because of tissue manipulation or processing. With ORO staining and TEM, fewer but larger lipid droplets were mainly observed in the most superficial layers of the CRISPR 12R-LOX TES model than in the controls. Large droplets such as those seen in CRISPR-LOX TESs were also observed in skin samples from patients with a mutated form of ALOX12B in ARCI cases and in other forms of ichthyosis [10,60,61], as well as in Alox12b knockout mice [62]; however, their nature remained unclear. This suggests a different form of lipid accumulation in the granular layer or the SC of CRISPR-LOX TESs that may represent another sign that lipids are differently processed in LOX-deficient keratinocytes. As seen before, altered lipidic profiles may lead to impaired keratinocyte differentiation [63], which may provide an explanation for the altered SC observed in CRISPR-LOX TESs.

To our knowledge, the CRISPR 12S-LOX TES could be the first human skin model with a defective expression of the native form of the 12S-LOX protein. In that sense, no disease with a loss of function of 12S-LOX gene activity has been reported to date [64]. The CRISPR 12S-LOX TES shared many features with the CRISPR eLOX-3 and CRISPR 12R-LOX models such as a granular aspect at a macroscopic scale as well as a reduced SC and large lipid droplets. In contrast to the CRISPR eLOX-3 and CRISPR 12R-LOX TESs, the CRISPR 12S-LOX TES did not show a thicker epidermis, and filaggrin staining was markedly reduced. Low filaggrin expression in these samples suggests that keratinocyte differentiation may be affected differently by native 12S-LOX depletion. Reduced filaggrin levels in 12S-LOX CRISPR TES may be explained by possible lipid abnormalities or changes in relative humidity levels following 12S-LOX reduced expression. Indeed, filaggrin expression was shown to be influenced by such factors [65,66]. Low filaggrin expression following 12S-LOX depletion is in accordance with the increased TEWL described in Alox12 knockout mice [14]. Indeed, low levels of filaggrin, as well as an impaired SC, are known to be associated with a compromised skin barrier function [67,68]. As seen for the Alox12 knockout mice and the human epidermal cell line SCL-II [14,69], CRISPR 12S-LOX TES did not show significant changes in cell proliferation. However, no histological or ultrastructural defects such as the one described in this study were reported in the Alox12 knockout mice [14]. Such a difference in the phenotype could be due to interspecies variations or to in vitro culture conditions that could promote the emergence of such a phenotype [70]. Having shown that this enzyme plays a critical function in the keratinocyte differentiation, the CRISPR 12S-LOX TES model has the potential to provide further insights into the role of 12S-LOX in skin barrier function.

Based on a proven technology for the production of skin substitutes, CRISPR-LOX TESs has the advantage of being stable and of human origin. Some studies did use animal cells or the shRNA transfection of human keratinocytes [57,58] to study LOX function. According to our experience, shRNA transfection may not allow for a complete and long-term downregulation of LOX in the modified cells, as is the case for CRISPR-Cas9(D10A). Compared to the use of shRNA, the CRISPR-Cas9(D10A) technique has the advantage of easily allowing for a specific and longer protein depletion within the cells [71], thus facilitating research on long-term processes such as keratinocyte differentiation. Complete keratinocyte differentiation in the CRISPR-LOX TES also facilitates the study of the role of these enzymes in human epidermis maturation. Globally, such models show advantages that could be useful in protein function studies.

## 5. Conclusions

The CRISPR-LOX TES models produced within this study successfully reflected the effects of eLOX-3 or 12R-LOX depletion, thus confirming the reliability of the model. The CRISPR 12S-LOX model also revealed a role for 12S-LOX within the keratinocyte differentiation process. The CRISPR-LOX TES model can be useful for the study of diseases or conditions that affect the epidermis and in which LOXs are suspected to be involved, such as ARCI, wound healing, photodamage, and atopic dermatitis. Using the CRISPR-Cas9(D10A) method described here, TESs can also be used to produce other models with reduced protein expression. In that sense, TESs have the advantage of being of human origin, as well as being reliable, ethical, and flexible models to which various elements can be added [36,72] and different kinds of studies can be performed [73,74]. Such models appear promising for the advancement of knowledge in the field of dermatology or as a replacement of animal models.

## Data Availability

Data are available upon request to the corresponding author.

## Supplementary Materials

The following supporting information can be downloaded at https://www.mdpi.com/article/10.3390/dermatopathology12030030/s1. Material and Methods: Site-directed mutagenesis (Cas9-D10A production). Lentiviral vector production. Production of TES. Table S1: Sequences of ssDNA inserted in the cassettes for sgRNA production and the associated specificity scores and off-target predictions for each sgRNA in CRISPOR. Table S2: Possible off-target genes (predicted using CRISPOR). Table S3: Adapter and primers used for sequencing following Golden Gate assembly and for the PCR amplification of lipoxygenase genes. Table S4: List of antibodies used for immunofluorescence staining. Figure S1: ICE results for the keratinocytes transduced with CRISPR-eLOX3 vs. non-targeting sgRNA control used for CRISPR-TES production. Figure S2: ICE results for the keratinocytes transduced with CRISPR 12R-LOX vs. non-targeting sgRNA control used for CRISPR-TES production. Figure S3: ICE results for the keratinocytes transduced with CRISPR 12S-LOX vs. non-targeting sgRNA control used for CRISPR-TES production.

## Abbreviations

The following abbreviations are used in this manuscript:

ARCI	Autosomal Recessive Congenital Ichthyosis
12R-LOX	12R-Lipoxygenase
12S-LOX	12S-Lipoxygenase
CLE	Cornified Lipid Envelope
CRISPR	Clustered Regularly Interspaced Short Palindromic Repeats
eLOX-3	Epidermis-type lipoxygenase 3
GFP	Green Fluorescent Protein
NCIE	Non-bullous Congenital Ichthyosiform Erythroderma
ORO	Oil Red O
SC	*Stratum Corneum*
TEM	Transmission Electron Microscopy
TES	Tissue-Engineered Skin
TEWL	Transepidermal Water Loss
