# Supplementary material for: Mimicking the LOX-Related Autosomal Recessive Congenital Ichthyosis Skin Disease Using a CRISPR-Cas9 System and Unravelling 12S-LOX Function in the Skin
Source: Dermatopathology (Basel). 2025 Sep 11;12(3):30. doi: 10.3390/dermatopathology12030030 (PMC12452335; doi:10.3390/dermatopathology12030030)

Supplementary material

Mimicking the LOX-related Autosomal Recessive Congenital Ichthyosis Skin Disease Using a CRISPR-Cas9 System and Unraveling 12S-LOX Function in the Skin.

Carolyne Simard-Bisson [a], Sébastien Larochelle [a], Véronique J. Moulin [a,b]*, Bernard Fruteau de Laclos [a,c]*

*: co-senior authors

[a] Centre de recherche du CHU de Québec-Université Laval and Centre de recherche en organogénèse expérimentale de l'Université Laval/LOEX, Québec, Québec, Canada

[b] Department of Surgery, Faculty of Medicine, Université Laval, Québec, Québec, Canada

[c] Department of Molecular Biology, Medical Biochemistry and Pathology, Faculty of Medicine, Université Laval, Quebec City, Québec, Canada.

*Correspondence: veronique.moulin@fmed.ulaval.ca

1. Materials and methods

1.1 Site-directed Mutagenesis (Cas9-D10A production)

The pLV hUbC-Cas9(D10A)-T2A-GFP plasmid was constructed by digesting pLV hUbC-Cas9-T2A-GFP (gift from Charles Gersbach, Addgene plasmid # 53191; http://n2t.net/addgene:53191; RRID: Addgene_53191) with XbaI (New England Biolabs, Ipswich, Massachusetts, #R0145S) and XcmI (New England Biolabs, #R0533S), treating with CIP (New England Biolabs, #R0525S), purifying the 14 kb fragment on a 0.5% agarose gel (QIAquick Kit, Qiagen, Germantown, Maryland, cat. # 28104) and by

quantifying the DNA by spectrometry (Nanodrop, Thermo Fisher Scientific, Waltham, MA, USA). A megaprimer [26] was generated by PCR using 10 ng pLV hUbC-Cas9-T2A-GFP, Q5 hot start polymerase (New England Biolabs, #M0493S), 10 mM dNTPs (Bio Basic, Markham, Ontario, Canada, #DD0058), 10 µM Cas9 Fwd ext XbaI primer (GTCGACTCTAGAGCCACCATGGACTAC) and 10 µM Cas9 D10A 4519C rev primer (CGCTGTTTGTGCCGATAGCGAGCCCAATGGAGTACTTCTTG).

Thermocycling conditions were as follows: an initial denaturation at 98 °C for 30 seconds, followed by 35 cycles at 98 °C for 5 seconds, 67 °C for 15 seconds, and 72 °C for 20 seconds, with a final extension at 72 °C for 2 minutes. The PCR product (megaprimer) was purified on an EZ-10 Spin Column (Bio Basic) and quantified by spectrometry. A second PCR was performed under similar conditions using the megaprimer and the Cas9 Rev XcmI reverse primer (CGTCCACGATATTGCCAAAG). The primers used were synthetised by Integrated DNA Technologies (Coralville, Iowa). The final product was purified and digested with XbaI, XcmI and EcoRV (New England Biolabs, #R0195S). The purified digested Cas9 D10A insert was ligated overnight using T4 DNA ligase (New England Biolabs, #R35395) with the linearised, CIP-treated vector. The resulting constructs were amplified using One Shot™ Stbl3 chemically competent E. coli (Invitrogen, Waltham, Massachusetts, #C737303) following the manufacturer's protocol. Clones were selected on LB agar (Sigma-Aldrich, St-Louis, MO, #L7025) with 100 µg/mL carbenicillin (Bio Basic, #CDJ469). Following the selection and amplification of positive colonies, plasmid DNA was extracted using the GenElute HP plasmid miniprep Kit (Sigma, #NA0160). The loss of an EcoRV site and the presence of the D10A mutation

were confirmed by digestion and sequencing, respectively, to verify successful vector construction.

1.2 Cell Culture

Fibroblasts derived from the breast skin biopsy of an 18-year-old adult woman were isolated as previously described [27,28]. After isolation, the cells were cultured in fibroblast culture medium as previously described [38] except that fetal calf serum was replaced by fetal bovine essence (FBe; Avantor). Human keratinocytes were isolated from the facelift resection of a 56 year-old woman as previously described [27] except for the thermolysin incubation that was performed for 18 hours at 4 ˚C. Keratinocytes were then cultured on a feeder layer of irradiated human fibroblasts in keratinocyte medium made of Dulbecco's modified Eagle's medium with Ham's F-12 medium in a 3:1 proportion (Gibco, #21700) supplemented with 24.3 mg/L adenine (Sigma-Aldrich, #A2786), 5% Fetalclone II serum (Hyclone, Logan, Utah), 5 µg/mL insulin (Sigma-Aldrich, #I5500-1G), 0.4 µg/mL hydrocortisone (Galenova, St-Hyacinthe, Québec, Canada #3HY220-005), 0.212 µg/mL isoproterenol (Sigma-Aldrich, I5627-5G), 10 ng/mL epidermal growth factor (Ango/Austral Biologicals, San Ramon, California, #GF-0108), 100 IU/mL penicillin (Sigma-Aldrich, #P3032) and 25 µg/ mL gentamicin (Gemini-Bio, West Sacramento, California, #400-100P). The cell culture medium was changed three times a week and keratinocytes passaged before confluency. According to previous studies, discrepancy in the age or in the origin of the fibroblasts does not have a significant impact on skin phenotype and expression profiles ([29,30] and data to be published).

1.3 Lentiviral vector production

HEK293FT cells were seeded in 6 well-plates coated with poly-D-lysine bromide (Sigma #P7280). The next day, the plasmid of interest, the envelope protein expressing vector pMD2.G (pMD2.G was a gift from Didier Trono, Addgene plasmid # 12259; http://n2t.net/addgene:12259; RRID:Addgene_12259) and the packaging protein expressing vector psPAX2 (psPAX2 was a gift from Didier Trono, Addgene plasmid # 12260; http://n2t.net/addgene:12260; RRID:Addgene_12260) were mixed in Opti-MEM (Gibco, Grand Island, New York #31985062) with 0.1% Fetal Bovine Essence (FBe, Avantor, Radnor, Pennsylvania) and Lipofectamine 2000 (Invitrogen, #100014469). HEK293FT cells were transfected by removing the cell culture medium and adding 500 µL of the plasmid-lipofectamine mix. Four hours later, 650 µL of Opti-MEM supplemented with 30% FBe was added to the cells. After 16 hours, the Opti-MEM was replaced with fibroblast cell culture medium (see the Cell culture section for details about the media). For the next three days, supernatants containing lentiviral vectors were harvested every twelve hours and placed at 4 ˚C. At the end of the three days, lentiviral vector harvests were filtered on a 0.45 µm filter and centrifuged at 50 000 g for 90 minutes at 4 ˚C. Lentiviral vectors were resuspended in fibroblast medium and incubated overnight at 4 ˚C under agitation. Lentiviral suspension was centrifuged again at 10 000 g for 5 minutes and supernatants were harvested and frozen at -80 ˚C.

1.4 Production of Tissue-Engineered Skin

Fibroblasts were grown in 12-well plates in DMEM +10% Foetal Bovine Essence supplemented with 50 µg/mL ascorbic acid (Sigma-Aldrich, St-Louis, MO, #A7631) for at least 24 days, thus allowing the production of extracellular matrix and the formation of

dermal sheets. Transduced or non-transduced keratinocytes were then seeded on top of half of the dermal sheets (25 000 keratinocytes/cm2) in 1 mL of keratinocyte culture medium. These sheets were kept in culture for 4 days in keratinocyte medium supplemented with 50 µg/mL ascorbic acid. Then, one dermal sheet seeded with keratinocytes was clipped onto a second dermal sheet to produce TESs. To induce keratinocyte differentiation, tissues were placed at the air–liquid interface for 14 days and cultured in keratinocyte culture medium deprived of Epidermal Growth Factor and supplemented with 50 µg/mL ascorbic acid. During the entire process, culture media were refreshed three times a week.

Table S1: Sequences of ssDNA inserted in the cassettes for sgRNA production and the associated specificity scores and off-target predictions for each sgRNA in CRISPOR

Target	Cassette	ssDNA oligonucleotides	Specificity score	Off-target number near PAM for 0-1-2-3-4 mismatches
Non targeting sgRNA control	H1	TCCCAGATCCATGTAATGCGTTCGA<br>AAACTCGAACGCATTACATGGATCT	N.A	N.A.
	7SK	CCTCGGTTCGACTCGCGTGACCGTA<br>AAACTACGGTCACGCGAGTCGAACC	N.A	N.A.
eLOX-3	H1	TCCCATGTCCAGTGTGCCGGCCCTC<br>AAACGAGGGCCGGCACACTGGACAT	90	0-0-0-0-0
	7SK	CCTCGGTCACACTGGTGGGCACGTG<br>AAACCACGTGCCCACCAGTGTGACC	86	0-0-0-1-2
12R-LOX	H1	TCCCACCACCATGTCGTCTGTGACG<br>AAACCGTCACAGACGACATGGTGGT	87	0-0-0-0-0
	7SK	CCTCGCGAGGGAACGTGCTTGCAAG<br>AAACCTTGCAAGCACGTTCCCTCGC	94	0-0-1-0-1
12S-LOX	H1	TCCCACAGGGCCCTGCACCGTGATG<br>AAACCATCACGGTGCAGGGCCCTGT	86	0-0-0-0-1
	7SK	CCTCGCCGTGCTACCGCTGGGTGCA<br>AAACTGCACCCAGCGGTAGCACGGC	89	0-1-0-2-0

N.A. : Non applicable

Table S2: Possible off-target genes (predicted using CRISPOR) and showing mismatch less than 4 bp.

Targeted LOX	Gene	zone	NCBI summary (function)	Number of mismatch
eLOX-3	LOC105375930	intron	uncharacterised	3
	NXN nucleoredoxin	intron	This gene encodes a member of the thioredoxin superfamily, a group of small, multifunctional redox-active proteins. Members of this family are characterised by a conserved active motif called the thioredoxin fold that catalyses disulfide bond formation and isomerisation. The encoded protein acts a redox-dependent regulator of the Wnt signaling pathway and is involved in cell growth and differentiation.	3
12R-LOX	LOC107985675	intergenic	ncRNA	2
12S-LOX	LOC124903908	exon	ncRNA	1
	IFFO2 intermediate filament family orphan 2	intron	Predicted to be located in intermediate filament.	2
	GRHL3-AS1 GRHL3 antisense RNA 1	exon	ncRNA	2

Table S3: Adapter and primers used for sequencing following Golden Gate assembly and for the PCR amplification of lipoxygenase genes

Target	Primers' sequence
DNA adapter for Golden Gate assembly	GATAGTAGCGAACGTGTCCGGCGT (FWD)<br>CAGGACGCCGGACACGTTCGCTAC (REV)
Primers for Golden Gate assembly sequencing	TCGGGTTTATTACAGGGACAGCAG (FWD)<br>TCTAAGGCCGAGTCTTATGAGCAG (REV)
Primers for eLOX-3	TCCAGGAAAGACAGGGACCTC (FWD)<br>CATCTCAGCATCTGATCCCTCC (REV)
Primers for 12R-LOX	CCT TGG GAA TCT GGT CTG GC (FWD)<br>CAAATGTCTCGTTGGGGTTGG (REV)
Primers for 12S-LOX	CAGTGACCCAGAGCCAATGT (FWD)<br>CTCTAAGTCTGCTCCGACCG (REV)

Table S4: List of antibodies used for immunofluorescence staining (NA: not available)

Antigen	Host	Supplier	Cat. number
eLOX-3*	Rabbit	Medimabs	NA
12R-LOX*	Rabbit	Peter Krieg [39]	NA
12S-LOX*	Rabbit	Novus	NBP-90338
Alexa 594 anti-rabbit	Chicken	Invitrogen	A21442
Transglutaminase 1	Rabbit	Protein tech	12912-3
Alexa 488 anti-rabbit	Goat	Invitrogen	A11034
Filaggrin	Mouse	Santa Cruz Biotechnology	SC-66192
Alexa 488 anti-mouse	Goat	Invitrogen	A11001
Ki67	Rabbit	Abcam	Ab15580

*** Antibody for which specificity was previously addressed [38].**

Figure S1: ICE[35] results for the keratinocytes transduced with CRISPR-eLOX3 vs non-targeting sgRNA control used for CRISPR-TES production

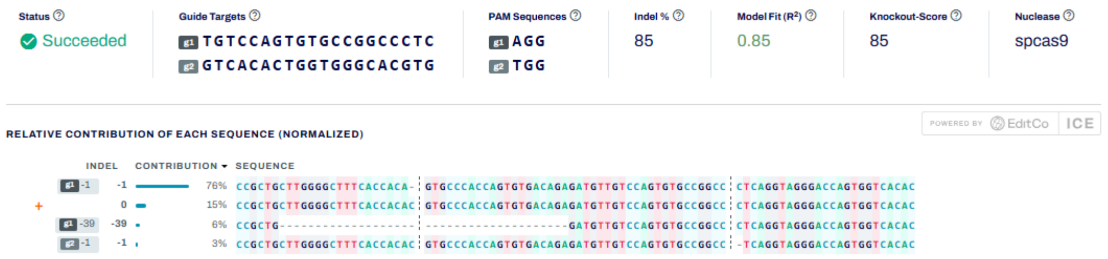

Figure S2: ICE[35] results for the keratinocytes transduced with CRISPR 12R-LOX vs non-targeting sgRNA control used for CRISPR-TES production

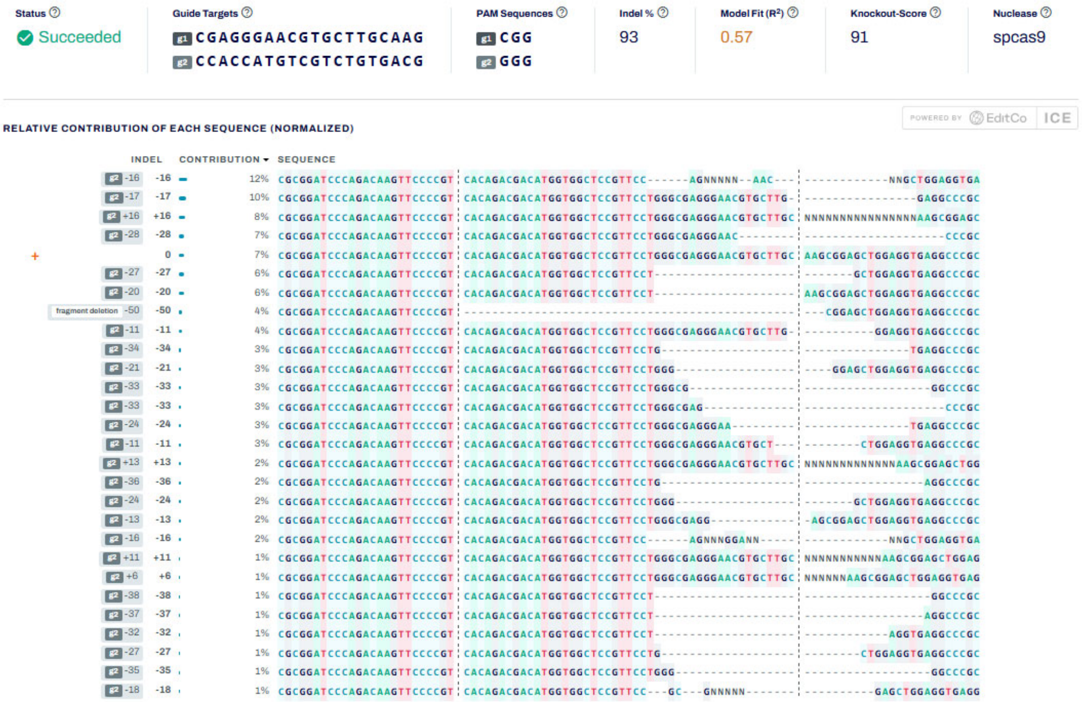

Figure S3: ICE[35] results for the keratinocytes transduced with CRISPR 12S-LOX vs non-targeting sgRNA control used for CRISPR-TES production

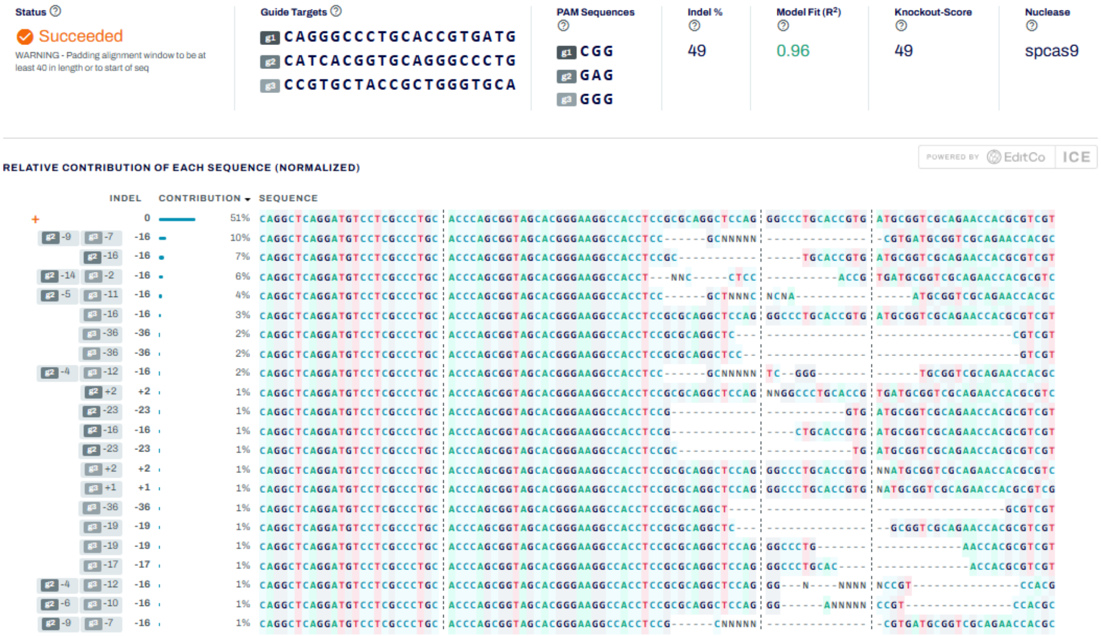

Supplement: Supplementary file 1 [file dermatopathology-12-00030-s001.zip › dermatopathology-3789496-supplementary.pdf]
